# Implementation of a patient-teaching workshop to improve pharmacy students’ competencies in patient-centered communication: a case report

**DOI:** 10.1186/s12909-022-03618-x

**Published:** 2022-07-18

**Authors:** Caroline Hache, Stéphane Honoré, Guillaume Hache

**Affiliations:** 1 Aix Marseille Univ, ADEF, 57 avenue escadrille Normandie Niemen, Marseille, 13013 France; 2 Aix Marseille Univ, Faculté de Pharmacie, Laboratoire de Pharmacie Clinique, 27 boulevard Jean Moulin, Marseille, 13005 France; 3 Aix Marseille Univ, AP-HM, Hôpital de La Timone, Service de Pharmacie, 264 rue saint Pierre, Marseille, 13005 France; 4 Aix Marseille Univ, Faculté de Pharmacie, 27, boulevard Jean Moulin, 13005 Marseille, France

**Keywords:** Patient-led education, Pharmacy, University curriculum

## Abstract

**Background:**

The pharmacist-patient relationship has evolved over recent decades and the development of clinical pharmacy requires pharmacists to take patient-centered responsibilities. This requires a specific set of skills, such as patient-centered communication. Evaluation of students’ competencies in patient-centered communication is challenging in academic settings and complementary assessment methods may be designed in order to overcome the limits of traditional preceptors’ ratings or objective structured clinical examination (OSCE). There is increasing interest in a more active patient role in healthcare professional education and there are very few reports about patient-led education in pharmacies. Thus, the objective of this work was to implement a patient-teaching workshop and to assess its impact on pharmacy students’ competencies in patient-centered communication.

**Methods:**

The workshop was developed in collaboration between four patients, a senior clinical pharmacist and a lecturer in education sciences and implemented in the hospital pharmacy residency program. The main course objective was acquiring the three competencies of the Calgary-Cambridge guide to the medical interview: (i) building a relationship, (ii) conducting structured interview and (iii) gathering information. The learning process integrated: working on participants’ perception of pharmacists-patient communication, a first simulated interview, didactic learning and a second simulated interview. After simulated interviews, patients and peer residents assessed learner’s performance with a competency chart and provided individual feedback. Assessment methods included comparisons between the first and second interview scores and an anonymous post-course survey.

**Results:**

Forty-seven residents and 19 patient teachers attended the session. Competency scores were higher after the second interview in all three competencies as rated by both patients (+ 25%) and peer residents (+ 29%).

Residents expressed a high satisfaction and reported learning about conducting interviews and soft skills contributing to the development of a relationship with patients. “The involvement of patients” was expressed as most appreciated in the majority of the evaluation charts (87%) and the residents valued the importance of collaborative and interprofessional learning during the workshop. Three themes emerged: (1) patients’ expertise, (2) reliability and (3) relationship, which underlined that the students estimated the patients were credible sources of information in this pedagogical context.

**Conclusion:**

This patient-teaching approach improved patient-centered competencies of pharmacy residents and promoted partnership between patients and pharmacy students.

## Background

Improving communication between healthcare professionals and patients is associated with better clinical outcomes [1]. The pharmacist-patient relationship has changed over recent decades and the development of clinical pharmacy allows pharmacists to take patient-centered responsibilities [2]. Pharmaceutical care involves therapy and decisions about the use of medication for a patient. During the process, the pharmacist co-operates with a patient and other professionals in designing, implementing, and monitoring a therapeutic plan in order to produce specific therapeutic outcomes for a patient [3]. Patient-centered care requires a specific set of skills and training, such as patient-centered communication and conducting structured interviews.

Evaluation of students’ competencies is challenging in academic settings and remains controversial in pharmacy practice experiences. In particular, assessments of student performance by traditional preceptors have been criticized for their lack of consistency and accuracy [4]. In this context, the objective structured clinical examination (OSCE), initially introduced in medical curricula, has been adapted to pharmacy curricula. OSCEs allow to assess clinical competencies and soft skills, such as professionalism and communication, during simulated situations. OSCEs may be used as formative and summative measures throughout the Pharmacy program. Compared to other assessments, the OSCEs have relatively high reliability, validity, and objectivity. However, the idealized textbook’ scenarios of OSCEs may not mimic real life situations and the precision of standardized patient simulation may influence student performance [5, 6]. Thus, complementary assessment methods may be designed in order to overcome these limits.

There is growing awareness and promotion for public and patients’ involvement in education of health care professionals, and professionals in training are in favor of a more direct patient involvement with their teaching [7]. Patient-led education is the active involvement of real patients in professional training, portraying their own experience of health care. It contributes to the implementation of practical experience of working with patients during training, in addition and complementarily to the theoretical and scientific aspects of health care providers’ education [8]. Surprisingly, there are few reports about patients’ involvement in pharmacist training, and most often patients were involved passively, as objects in case studies or bedside teaching. There is increasing interest in a more active patient role in healthcare professional education, as it may help to enhance student experiences of their future professional settings by recognizing patients as “experts” in their own medical conditions [9]. There is a wide range of degrees in the extent to which patients are actively involved in the education of healthcare professionals and many variables in how they are involved. The continuum of patient engagement in education has been conceptualized and developed at the University of Montreal, so called “the Montreal model”, from information to full partnership in which patients and faculty members co-construct the programs and co-teach during the sessions [10]. In addition, Towle et al. proposed a taxonomy to characterize the degree of involvement which may clarify the patient’s roles [11]. For example, patient-teachers are involved in educational delivery, development and evaluation.

Thus, the aim of our work was to develop a workshop about patient-focused communication, involving patient-teachers and to assess its impact on learners’ competencies in communication.

## Methods

The study covered a module focused on clinical pharmacy during the residency program in hospital pharmacy.

### Development and format of the workshop

The workshop was developed in collaboration between four patients, a senior clinical pharmacist and a lecturer in education sciences. The clinical pharmacist and the lecturer in education sciences designed a pilot educational device. This pilot was submitted to four patients recruited in the University expert patient program. The general structure of the workshop was appreciated. We agreed that during the workshop, the leadership would be shared between the clinical pharmacist and the patients. Notably, patients would independently conduct the activity-based learning described below. We have added elements based on patients’ recommendations, such as a dedicated time for student feedback on their involvement during the workshop and providing standardized tools to run the role play.

The main course objective was acquiring the three competencies of the Calgary-Cambridge guide to the medical interview: (i) building a relationship, (ii) conducting a structured interview and (iii) gathering information [12]. The workshop lasted four hours and consisted of four main steps.

- Step 1: we started the session with the statements about the need for confidentiality and respect, then a round of introductions. We announced and explained the competencies framework in order to build the learning agreement and illustrate the behaviors expected by the faculty. Then, a 15-min brainstorm on pharmacist-patient communication was designed as an icebreaker in the group, involving residents and patients.
- Step 2: a role play was implemented, simulating a medication history interview. The patient teachers played the role of patient during the simulation and then provided feedback on the way they perceived the interview to the resident.
- Step 3: didactic learning about theoretical aspect of patient-centered communication was provided by the clinical pharmacist supported by a slide presentation and illustrated by the patients’ experiences.
- Step 4: a second role play simulating a medication history interview was performed. Patients met a different resident than in the first performance. Individual feedback was provided by patient teachers and finally a collective feedback was organized.

Patient teachers were recruited on a voluntary basis, from the Alumni network of the local University of patients and were all graduates of the University expert patient program. For the purpose of the workshop, volunteers were individually briefed about the background of the residents and the format of the course. No scenario was given in order to promote spontaneity and realism during the role plays and debriefing.

### Assessment and measures

After simulated interviews, patients and peer residents assessed learner’s performance with a competency chart. This competency chart was inspired from the Calgary-Cambridge guide to the medical interview [12] and contained 14 skills to be assessed; six in the competency (i) building a relationship, four in the competency (ii) conducting a structured interview and four in (iii) gathering information. Each skill was rated from 1 (non-acquired) to 5 (mastered). Scores for each competency were combined to a 100-point total in order to make the results practical to compare and interpret across dimensions. Score comparisons have been assessed by a Wilcoxon-Mann–Whitney test and correlations estimated by the Pearson correlation coefficient.

In addition, we evaluated satisfaction, metacognition and self-efficacy by a post-course survey with open-ended questions in order to estimate the impact of the session. Open-ended responses were analyzed through a content analysis process, in order to describe and categorize common words, phrases and ideas in qualitative data.

The pedagogical committee of Aix-Marseille University-School of Pharmacy that deals with research authorizations and ethical considerations in the field of education has approved the study. Verbal consent was obtained from study participants and approved by the committee. The ethical considerations taken into account were based on the principles outlined in the Declaration of Helsinki.

## Results

**Fig. 1:**
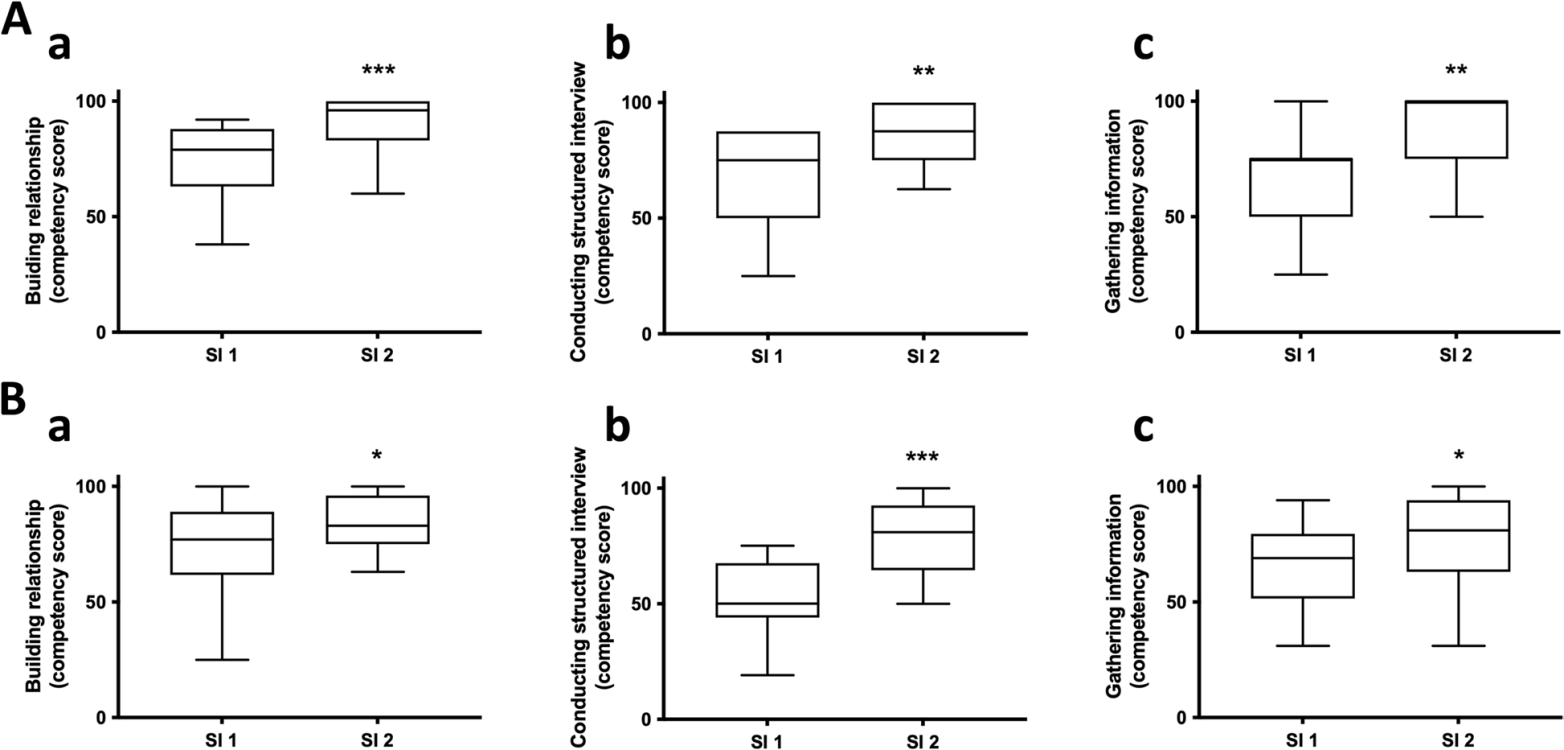
Acquisition of communication competencies, assessed by patient teachers and peer residents. Comparison of competency scores assessed by patients (**A**) and peers (**B**), in the three competencies *conducting structured interview* (**a**)*, building relationship* (**b**) and *gathering information* **(c**) between simulated interview 1 (SI 1) and simulated interview 2 (SI 2). Data are plotted as box and whister (min to max; line at median). *p < 0.05; **p < 0.01; ***p < 0.001

Forty-seven pharmacy residents attended the session, nine men and 38 women who were between 22 and 25 years old. In addition, 19 patients participated in the workshop as patient teachers, including the four patients involved in the design. Patients evaluated higher competency scores after the second interview than the first in all competencies ie (i) building a relationship (91.6 ± 11.1 vs 74.5 ± 15.5; *p* < 0.001), (ii) conducting structured interview (87.5 ± 12.5 vs 69.7 ± 19.7; *p* = 0.005) and (iii) gathering information (82.9 ± 20.5 vs 65.8 ± 19.0; *p* = 0.01). Peer residents evaluated higher competency scores after the second interview than the first in all competencies ie (i) building a relationship (83.7 ± 12.6 vs 72.0 ± 20.5; *p* = 0.036), (ii) conducting structured interview (79.4 ± 15.1 vs 51.2 ± 16.6; *p* < 0.001) and (iii) gathering information (76.9 ± 20.6 vs 66.6 ± 16.3; *p* = 0.030) (Fig. 1). Competency scores assessed by peer residents after the second interview were significantly correlated to scores assessed by patients in all competencies ie (i) building a relationship (*r* = 0.706; *p* < 0.001), (ii) conducting structured interview (*r* = 0.673; *p* < 0.001) and (iii) gathering information (*r* = 0.413; *p* = 0.032). These results illustrated the short-term acquisition of competencies.

We obtained 47 responses from residents to the survey. They expressed a high general satisfaction, valued the collaborative environment and the importance of interprofessional learning during the workshop. They reported learning about expertise on how to conduct an interview and soft skills related to professional posture in the relationship with patients. This is a metacognitive approach of learning. Finally, they estimated to be able to and reported willingness to implement new skills in professional settings, which illustrates self-efficacy (Table 1).

**Table 1:** Exemplary quotes representing themes from qualitative analysis of student reflections on the workshop through pharmaceutical interview course learning objectives in open-ended survey responses

**Satisfaction**	**Value of the collaborative environment** - “I appreciated the spontaneity of exchanges.” - “I loved learning from patients without ego, without the stress of hospital settings.” - “I appreciated the freedom of speech.”
	**Importance of interprofessional learning** - “The patients’ perception on my interview and feedback were much instructive.” - “We learned much more than only theory.”
**Metacognition**	**Expertise on how to conduct an interview** - “I’ve learnt how to conduct a structured interview with patients.” - “How I can better formulate open questions, announce the sessions and introduce myself.”
	**Soft skills related to professional posture in the relationship** - “Active listening to what patients have to say.” - “ The importance of non-verbal communication for patients (smile, attitude, empathy).”
**Self efficacy**	**Implementation of skill in professional settings** - “I’ll try, in the future, to implement the communication techniques learnt today, when performing pharmaceutical interviews.” - “I will implement the structure of the interview, focusing on patients’ personal life, without going into details; letting the patient talk.”

“The involvement of patients” was expressed as most appreciated in the majority of the evaluation charts (87%) and valued this involvement as a highlight of the session. The verbatim were categorized in three themes. First, residents acknowledged patients’ expertise in their relationship with healthcare professionals:

- “I’ve learned to transfer patient experience reports into practices
- I appreciated discussions about the patients’ perspective on their relationship to pharmacists and their role in healthcare
- I will use advice given by patients on what can be done or what must be avoided during a pharmaceutical interview “

Then, they estimated patients’ perspective as “true”, reliable:

- “I loved to listen to a true point of view
- I loved talking with true patients who have concrete things to share
- I appreciated the exchanges on real life experiences”
  Finally, they developed confidence in the relationship they built with the patients during the workshop.
- “I appreciated proximity to patients and their feedback
- I loved freedom of speech / the opportunity to ask all our questions
- Everyone was free to express their feelings, no stereotyped language”

## Discussion

We describe a competency-based education workshop in Pharmacy curriculum, focused on three transversal competencies to conduct a pharmaceutical interview, and involving patients as partners. Our results demonstrated the positive impact of the workshop on residents’ perspectives in the pharmacy training program. First, the workshop was well received by the residents who expressed a high level of satisfaction, highlighting the relevance of the workshop [13]. Notably, the collaborative environment and the interprofessional learning were highlighted as the most appreciated aspects of the workshop. This is a finding consistent with most other reports about patient involvement [11–14]. Secondly, the residents expressed learning outcomes about how to conduct an interview and the professional posture to promote the relationship. The residents reported that they acquired knowledge about the three main learning objectives of the workshop, which is a metacognitive approach of learning. Metacognition is the awareness of intellectual processes implemented to earn and it contributes to the acquisition of knowledge [15]. Finally, all participants reported skills to implement which illustrates learning outcomes. In addition, they reported the intention to set up these skills in their professional context, in order to improve communication. These results suggest that residents were not reluctant to changes, and that they felt able to improve their behaviors. Perceived self-efficacy is defined as people’s judgements of their capacities to organize and execute a course of action required to attain designated types of performances [16]. Interestingly, self-efficacy has been identified as a motivational parameter involved in both adoption of and long-term adherence to these behaviors, and has a positive impact on work-related performance [17, 18]. Thus, we suggest that our results are promising for long-term skill persistence. Taken together, relevance, knowledge acquisition and competency met three levels of the Kirckpatrick assessment method [13].

The design of our study did not allow to assess performance in a professional context on a long-term basis. However, we measured an increase in competency scores over the course of the session, which demonstrated short-term skill acquisition and behavior change. Interestingly, behavior change was evaluated by both the patients and the peer residents. Since there is no gold standard for measuring the development of a relationship, multi-perspective assessment appears the best approach [19]. In our setting, patient assessment may be interpreted as patient satisfaction in learning and provides the external validation of the learning effect. Notably, previous studies suggested that patient evaluation does not discriminate sufficiently among students, due to patients’ tendency to rate students favorably [20]. Thus, we associated rating by peer residents. Peers are aware of both academic and professional expectations, and previous work suggests that peer assessment tend to be correlated with teachers’ assessment of the same students [21–24].

In our study, patients as partners were free to portray their own case and/or improvise on the situation in order to promote the diversity. This concept forms the foundation of patient-centered care, and are concepts that students rarely learn in classes, where similarities rather than diversity among patients are emphasized [11, 25]. This was a step further in the realism of training compared to OSCE where the textbook’ scenarios may not mimic real life situations and the precision of standardized patients may influence the student [5, 6, 26]. In addition, the originality of our work was to involve patients as assessors of student competencies. After the assessment phase, individual feedback from patients to learners formed the cornerstone of the learning process. Patients were in a position of individual coaches, independent from the faculty, and messages transmitted by the patients were assessed as “true” by the residents. Importantly, we report that students acknowledged patients’ expertise in their relationship to professionals, attributed reliability to patients’ speech and developed confidence in the relationship they built with the patients during the workshop. The credibility of a speaker is defined as the degree to which a source of information is perceived as relevant, competent and reliable by the recipient of the message [27]. Expertise and reliability therefore appear as main dimensions of the issuer’s credibility. Thus, the residents estimated that the patients were credible sources of information in this pedagogical context [28]. Here, we underline that collaborating with patients during the pharmacy training program, especially in individual feedback and in the assessment process, contributes to generating trust and the acquisition of competency. Complementary with OSCEs, this kind of workshop represents a model that pharmaceutical schools can adapt in the objective to improve patient-centered competencies programs and promote partnership between patients and pharmacy students.

## Data Availability

The datasets used and/or analysed during the current study are available from the corresponding author on reasonable request.

## Abbreviation

OSCE: Objective structured clinical examination
